# Automated Detection of Sleep Apnea-Hypopnea Events Based on 60 GHz Frequency-Modulated Continuous-Wave Radar Using Convolutional Recurrent Neural Networks: A Preliminary Report of a Prospective Cohort Study

**DOI:** 10.3390/s22197177

**Published:** 2022-09-21

**Authors:** Jae Won Choi, Dong Hyun Kim, Dae Lim Koo, Yangmi Park, Hyunwoo Nam, Ji Hyun Lee, Hyo Jin Kim, Seung-No Hong, Gwangsoo Jang, Sungmook Lim, Baekhyun Kim

**Affiliations:** 1 Department of Radiology, Armed Forces Yangju Hospital, Yangju 11429, Korea; 2 Department of Radiology, Seoul Metropolitan Government—Seoul National University Boramae Medical Center, Seoul National University College of Medicine, Seoul 07061, Korea; 3 Department of Neurology, Seoul Metropolitan Government—Seoul National University Boramae Medical Center, Seoul National University College of Medicine, Seoul 07061, Korea; 4 Department of Otorhinolaryngology-Head and Neck Surgery, Seoul Metropolitan Government—Seoul National University Boramae Medical Center, Seoul National University College of Medicine, Seoul 07061, Korea; 5 AU Inc., Daejeon 34141, Korea

**Keywords:** obstructive sleep apnea, polysomnography, radar, deep learning, convolutional recurrent neural network

## Abstract

Radar is a promising non-contact sensor for overnight polysomnography (PSG), the gold standard for diagnosing obstructive sleep apnea (OSA). This preliminary study aimed to demonstrate the feasibility of the automated detection of apnea-hypopnea events for OSA diagnosis based on 60 GHz frequency-modulated continuous-wave radar using convolutional recurrent neural networks. The dataset comprised 44 participants from an ongoing OSA cohort, recruited from July 2021 to April 2022, who underwent overnight PSG with a radar sensor. All PSG recordings, including sleep and wakefulness, were included in the dataset. Model development and evaluation were based on a five-fold cross-validation. The area under the receiver operating characteristic curve for the classification of 1-min segments ranged from 0.796 to 0.859. Depending on OSA severity, the sensitivities for apnea-hypopnea events were 49.0–67.6%, and the number of false-positive detections per participant was 23.4–52.8. The estimated apnea-hypopnea index showed strong correlations (Pearson correlation coefficient = 0.805–0.949) and good to excellent agreement (intraclass correlation coefficient = 0.776–0.929) with the ground truth. There was substantial agreement between the estimated and ground truth OSA severity (kappa statistics = 0.648–0.736). The results demonstrate the potential of radar as a standalone screening tool for OSA.

## 1. Introduction

Obstructive sleep apnea (OSA) is the most common type of sleep-disordered breathing, characterized by the recurrent cessation of breathing during sleep due to complete or partial obstruction of the upper airway [1]. Repetitive episodes of hypopnea and apnea can result in sleep disruption and the alteration of neural activity due to intermittent hypoxemia, hypercapnia, microarousals, and fragmented sleep [2]. OSA is associated with an elevated risk of hypertension, stroke, and type 2 diabetes mellitus [3]. In addition, cognitive dysfunction, cardiovascular and cerebrovascular diseases, and increased mortality are highly prevalent in OSA patients [4,5]. Polysomnography (PSG) is the gold standard for diagnosing OSA [6]. However, PSG requires a specialized facility with trained technicians and various sensors directly attached to the patient’s body, which causes inconvenience and possibly affects sleep behavior [7]. The “first-night effect,” which has been considered a result of sleeping in the unfamiliar circumstance of a sleep laboratory, is one of the limitations of PSG [8]. In addition, because PSG innately evaluates only one or a few nights, it is inadequate for long-term monitoring and screening [9]. In this context, there has been an increasing interest in developing sensors from various sources with less or no contact, such as cameras, ballistocardiography, sound, and radar [10,11,12].

A radar is a non-contact detection system that processes radio waves transmitted to and reflected by a target. In the medical field, radar sensors typically analyze the modulation effect caused by body movements and have thus been developed for monitoring vital signs and limb movements [13,14]. Respiratory effort, which is conventionally measured with thoracoabdominal belts in PSG, can be detected using radar by targeting chest wall movements [15,16,17]. Compared with other non-contact sensors, radars can detect respiratory efforts at longer distances of up to 30 m, even in the presence of non-metal materials between the radar and the subject, making them more applicable to sleep environments [17]. There are different types of radar according to the type of transmitted signal, such as impulse radio ultra-wideband (IR-UWB) radar and frequency-modulated continuous-wave (FMCW) radar [13]. The present study utilizes an FMCW radar.

Previous studies have investigated radar-measured respiratory signals for diagnosing OSA using various interpretation methods, including manual reading [18], amplitude thresholding [19,20], and classical machine learning with handcrafted features [21,22,23]. However, these methods often rely on explicit rules and prior domain knowledge, making them less robust to dynamic and noisy real-world data [24]. In this regard, deep learning, a subset of machine learning and artificial intelligence, which has shown great success in medical imaging [25] and biosignals [26], is a promising alternative. However, deep learning studies on radar-based OSA diagnosis are rare, especially regarding FMCW radar.

Therefore, this study aimed to demonstrate the feasibility of applying deep learning to FMCW radar data for detecting sleep apnea-hypopnea events as a preliminary study of an ongoing prospective OSA cohort using an in-development radar. The main differences of this study compared to previous deep learning methods for OSA diagnosis are as follows: (1) we used a detection model with dense predictions instead of classification models, (2) we conducted an event-oriented and patient-oriented performance evaluation as well as segment analysis, and (3) we included the entire PSG recording without excluding waking hours. In particular, our deep learning method was based on a convolutional recurrent neural network (CRNN), which was first proposed for scene text recognition [27] and has also been used for sound event detection [28].

## 2. Materials and Methods

### 2.1. Study Participants

This study included participants from an ongoing prospective cohort study approved by the Institutional Review Board of the Boramae Medical Center of Seoul National University (IRB No. 10-2021-16) and was performed in accordance with the principles of the Declaration of Helsinki. All the participants provided written informed consent.

Participants were recruited from adults with chief complaints of sleep-disordered breathing, referred to the Boramae Medical Center of Seoul National University. The symptoms of sleep-disordered breathing include snoring, shortness of breath, and witnessed apnea during sleep. Participants with major medical conditions in the pulmonary, cardiovascular, or cerebrovascular systems were excluded. All enrolled participants completed sleep questionnaires and overnight PSG, and clinical information, including detailed sleep history, medical history, and body mass index, was obtained.

### 2.2. Sleep Questionnaires and Polysomnography

The participants were instructed not to drink alcohol or caffeinated beverages and to sleep and wake up at regular hours for a week before the study. Subjective daytime sleepiness was scored using the Epworth Sleepiness Scale [29] and the Stanford Sleepiness Scale [30]. Sleep quality and disturbances during the month prior to the study were evaluated using the Pittsburgh Sleep Quality Index [31]. The PSG recording was conducted with Twin-PSG software (Natus Neurology Incorporated, West Warwick, RI, USA) using a standard PSG routine with the addition of the radar sensor. The recordings of bioelectrical potentials included a 6-channel electroencephalogram, a 4-channel electrooculogram, an electromyogram, and an electrocardiogram. A thermistor, a nasal air pressure monitoring sensor, an oximeter, piezoelectric bands, and a body position sensor were also applied to the patients. PSG data, including sleep parameters and respiratory events, were scored according to the American Academy of Sleep Medicine (AASM) manual [32]. Apnea was defined as a ≥90% reduction in airflow lasting at least 10 s. Apnea was further classified as central or obstructive if inspiratory effort was absent or present, respectively, throughout the entire period of absent airflow. Hypopnea was defined as a ≥30% reduction in airflow lasting for at least 10 s and was associated with either a ≥3% oxygen desaturation or arousal. The apnea-hypopnea index (AHI) was defined as the number of apnea-hypopnea events divided by the total sleep time (hours) in the PSG scoring data. The severity of OSA was classified into four categories based on AHI: normal (AHI < 5), mild (5 ≤ AHI < 15), moderate (15 ≤ AHI < 30), and severe (AHI ≥ 30).

### 2.3. Radar Setup

**Figure 1:**
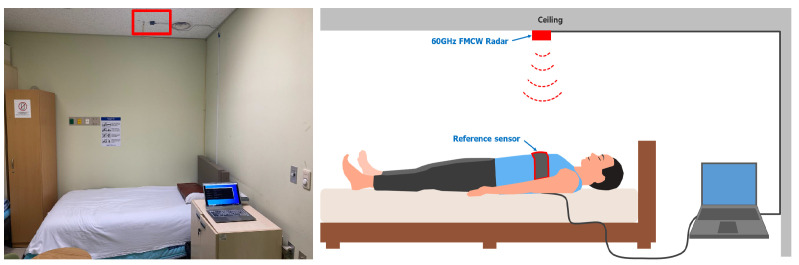
Photo and illustration of radar (red box) setup for respiratory signal monitoring.

**Figure 2:**
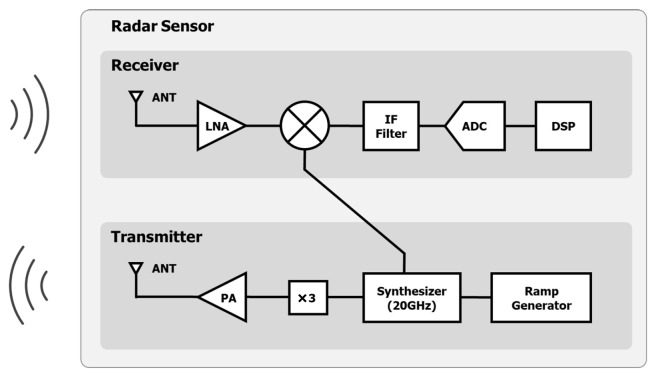
The architecture of the radar sensor: ANT = antenna, LNA = low-noise amplifier, PA = power amplifier, IF = intermediate frequency, ADC = analog-digital converter, and DSP = digital signal processor.

A FMCW radar sensor (AU Inc., Daejeon, Korea) was placed 2 m from the patient’s chest on the ceiling of the sleep laboratory (Figure 1). The architecture of the radar sensor is illustrated in Figure 2. The general principles of radar signal acquisition are provided in the Supplementary Materials.

**Figure 3:**
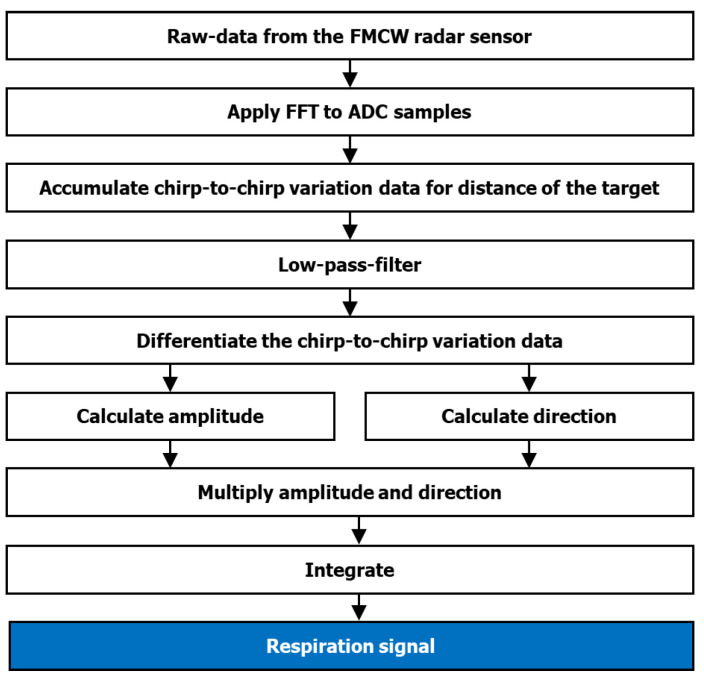
A flowchart describing the extraction of respiratory signals from radar signals.

Figure 3 shows the overall flow of how the respiratory signals were extracted from the raw radar signals. First, the distance to the target was determined by applying a fast Fourier transform to the ADC samples. Then, to remove high-frequency noise, chirp-to-chirp variation signals at the target distance were filtered using a low-pass filter. Finally, the respiratory signals were demodulated from the filtered signals.

**Figure 4:**
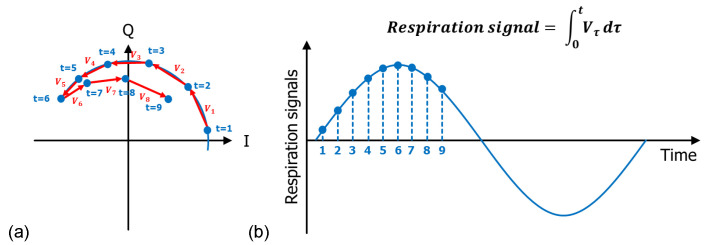
The mechanism for demodulating respiratory signals from radar output signals: (**a**) respiratory signals in the complex domain and (**b**) demodulated respiratory signal.

Most previous studies [33] used the phase demodulation method to extract respiration signals. The phase demodulation method suffers from the in-phase and quadrature (IQ) mismatch issues. The IQ mismatch changes the center coordinate of the trajectory, which is caused by respiration. In this study, we used the cumulative amplitude of a vector by subtracting two vectors in a complex domain for a unit of time, which corresponds to the time difference between chirps. Because our demodulation method considers the difference between the two vectors, the IQ mismatch effect can be suppressed [34]. Figure 4 shows the demodulation method. Respiratory signals in the complex domain are shown in Figure 4a. The respiratory signals were obtained by integrating the amplitudes of the vectors whose signs were determined by the direction. Figure 4b shows the demodulated respiratory signals.

### 2.4. Data Preparation and Preprocessing

A full-night radar recording was obtained regardless of sleep stages to emulate a real-world application, as the radar alone cannot distinguish the sleep status. The respiratory signals acquired from the radar were preprocessed using minimal signal-processing methods. The respiratory signals were first downsampled from the original 1000/33 Hz sampling frequency to 8 Hz using Fourier transformation. Next, the radar data were segmented into 1-min segments with a stride of 30 s. A segment-wise z-score normalization was then performed based on the mean and standard deviation of the signal values. Event labels were annotated with 1-s temporal resolution according to the reference PSG. Each segment was considered abnormal if it was labeled apnea or hypopnea for at least 10 consecutive seconds.

### 2.5. Model Development

A five-fold cross-validation was performed for model development and evaluation. First, the study population was randomly split into five groups. In each fold, the signal segments obtained from patients in the four subgroups served as the training set, and those from the remaining patients served as the validation set. In addition, two types of models were prepared: binary and multiclass. The apnea-hypopnea events were considered as a single abnormal class for the binary model and separated for the multiclass model.

Inspired by SELDnet [28] for sound event localization and detection, a detection model with a CRNN architecture composed of convolutional neural networks (CNNs), recurrent neural networks (RNNs), and fully connected (FC) components was employed. The CNN component consisted of four convolutional blocks. Each block was implemented as a stack of two sets of 1-D convolution, batch normalization, and rectified linear unit (ReLU) layers, followed by a max-pooling layer and dropout layer. All convolutional layers had 64 filters with a kernel size of 3 and a stride of 1. The max-pooling layers had a stride length of 2 except for the last block, which had a stride length of 1. The RNN component was a bidirectional long short-term memory (LSTM) layer with 128 units. The FC component consisted of an FC layer with 128 filters, a ReLU layer, a dropout layer, and a final FC layer with the same number of filters as the number of classes. All the dropout layers in the CRNN had a dropout rate of 0.2.

The sum of cross-entropy and Dice loss was used as the training objective adopted from medical image segmentation tasks [35] and a batch size of 64. The Adam optimizer [36] with an initial learning rate of 0.001 was used, which was reduced by half if the validation loss did not improve for 10 epochs. The maximum number of training epochs was set to 100, and early stopping was applied if the validation loss did not improve for 25 epochs. The model development was implemented using Keras (version 2.8.0; https://keras.io/) with a Tensorflow (version 2.8.0; Google LLC, Mountain View, CA, USA) backend on a workstation with an NVIDIA GeForce RTX 3080 GPU (Nvidia, Santa Clara, CA, USA) and 31 GB RAM.

### 2.6. Performance Evaluation

The performance evaluation was based on gathering inference results from the validation sets from each fold in the cross validation. Following the clinical significance of AHI, all apnea-hypopnea events were considered as a single abnormal class, and model predictions for each time point were calculated as 1−probabilityforclassNormal for both binary and multiclass models. Performance evaluation was conducted in three ways: per-segment classification, global event detection, and AHI estimation.

First, a receiver operating characteristic (ROC) curve was obtained for per-segment binary classification. Consistent with the ground truth labeling, the predicted probability of each 1-min segment was defined as the minimum threshold that yielded 10 consecutive seconds with above-threshold predictions. From the ROC curve, the area under the ROC curve (AUROC) was computed, along with 95% confidence intervals (CI) using the Delong method [37]. In addition, the optimal cut-off point yielding the maximum value of the Youden index [38] was obtained from the ROC curve. Based on the optimal cutoff point, the sensitivity, specificity, positive predictive value (PPV), negative predictive value (NPV), and accuracy were calculated.

In addition to the per-segment evaluation, the global event detection performance was assessed by computing the abnormal respiratory event detection sensitivity and false-positive detections per participant. The predictions from a patient’s full-night PSG were first aggregated as averages of prediction probabilities from two adjacent overlapping segments, because the segments were 1-min long with intervals of 30 s. Estimated abnormal respiratory events were defined as consecutive above-threshold predictions of at least 10 s and determined true-positive or false-positive at an intersection over union (IoU) threshold of 0.5 compared with the ground truth. The optimal cutoff point yielding the highest F1 measure, defined as the harmonic mean between precision and recall, was used to obtain the results [39]. Based on the optimal cut-off point, sensitivity, PPV, and false-positive detections per patient were calculated. In addition, since sleep status was not accounted for in the predictions, the estimated abnormal respiratory events were further categorized depending on whether they occurred during sleep or wakefulness. To this end, in-sleep estimated events were defined as events in which the patient was asleep for more than half of the event time.

Moreover, AHI was calculated as the number of estimated abnormal respiratory events divided by total sleep time and OSA severity and was estimated in line with the standard PSG scoring system [32]. Similarly, the corrected estimated AHI and OSA severity was calculated based on the number of in-sleep estimated abnormal respiratory events. The estimated AHIs were compared with ground truths using the Pearson correlation coefficient (*r*), intraclass correlation coefficient (ICC), and Bland–Altman analysis. The estimated OSA severities were compared with the ground truths using linear weighted kappa statistics (κ). The AHI estimation results were based on the same binarization thresholds used for event detection evaluation.

## 3. Results

### 3.1. Study Participants

Initially, 55 participants were enrolled in the study from July 2021 to April 2022. Among them, 11 were excluded from the study for the following reasons: withdrawal of consent (*n* = 9) and accidental failure to execute the radar data collection program (*n* = 2). Therefore, 44 participants underwent PSG integration using the FMCW radar sensor. Among them, there were nine normal participants, and the numbers of patients with mild, moderate, and severe OSA were 7, 15, and 13, respectively. The baseline demographic and sleep characteristics of the study population are presented in Table 1.

### 3.2. Per-Segment Classification Performance

**Figure 5:**
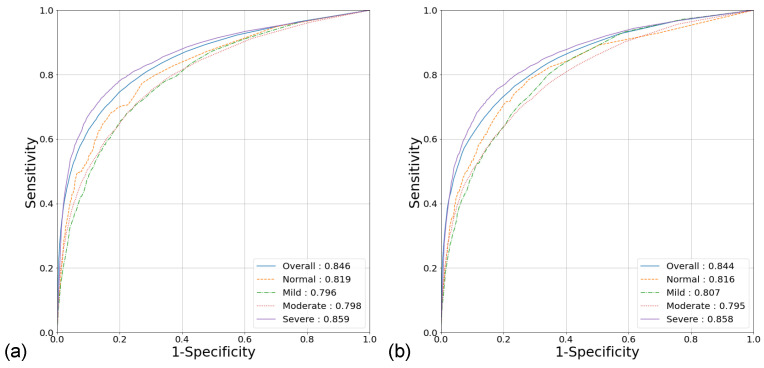
ROC analysis of per-segment classification for the (**a**) binary and (**b**) multiclass models.

The binary model showed an overall AUROC of 0.846 (95% CI [0.842, 0.851]) for per-segment classification, with a sensitivity of 74.4% (95% CI [73.6%, 75.3%]) and a specificity of 80.3% (95% CI [79.7%, 80.8%]). The multiclass model showed similar results, with an overall AUROC of 0.844 (95% CI [0.840, 0.849]), a sensitivity of 72.1% (95% CI [71.2%, 72.9%]), and a specificity of 81.3% (95% CI [80.8%, 81.8%]). Table 2 and Figure 5 show the detailed classification performance. In the OSA severity groups, the AUROC, sensitivity, and specificity ranged from 0.796 to 0.859, 62.5% to 80.9%, and 76.5% to 86.8%, respectively. The AUROC in patients with severe OSA was higher than that in the rest of the patients for both the binary (0.859 vs. 0.814, *p* < 0.001) and multiclass models (0.857 vs. 0.809, *p* < 0.001).

### 3.3. Global Event Detection Performance

The sensitivities of the binary and multiclass models were 63.3% (95% CI [62.1%, 64.5%]) and 62.2% (95% CI [61.0%, 63.4%]), respectively, for the overall study population, with a range of 49.0–67.6% across OSA severity. The number of false-positive detections per participant ranged from 23.4 to 52.8, and it was 17.2–31.0 when considering only estimated in-sleep events.

**Figure 6:**
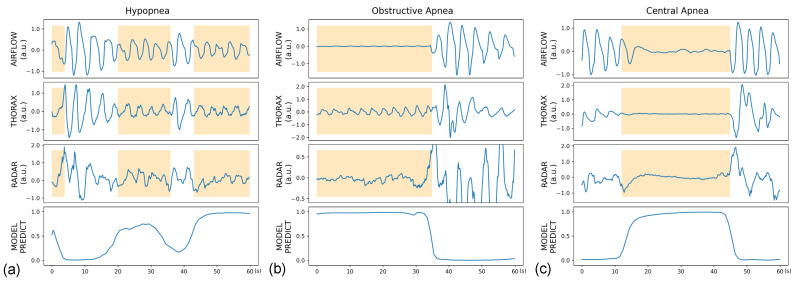
Representative cases of (**a**) hypopnea, (**b**) obstructive apnea, and (**c**) central apnea, presented along with predictions from the binary model. The ground truth events are annotated with orange boxes.

Moreover, per-class sensitivities in the overall study population ranged from 53.9% to 87.0%, with lower values for hypopnea than for apnea. The sensitivity for hypopnea across OSA severity was 47.6–58.1%, whereas the sensitivity for obstructive and central apnea showed a wide range of 0.0–100.0% due to the small number of apnea cases in non-OSA and mild OSA participants. In patients with moderate or severe OSA, the sensitivities for obstructive and central apnea were 86.1–92.0% and 78.9–88.0%, respectively. The detailed event detection performance is presented in Table 3. Figure 6 illustrates the representative cases of different respiratory events with the model predictions.

### 3.4. AHI Estimation Performance

**Figure 7:**
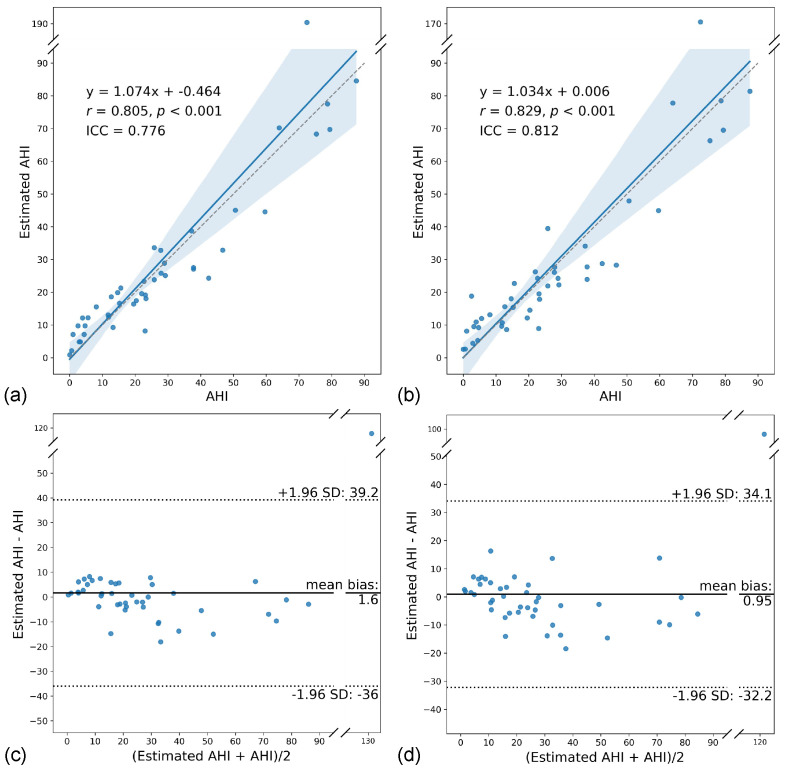
Comparison of the estimated AHI with the ground truth for the binary (**a**,**c**) and multiclass models (**b**,**d**). (**a**,**b**) Scatter plots of estimated AHI versus AHI showing the linear regression line (blue line) with 95% confidence intervals (blue shadowed area). (**c**,**d**) Bland–Altman plots of estimated AHI and AHI.

**Figure 8:**
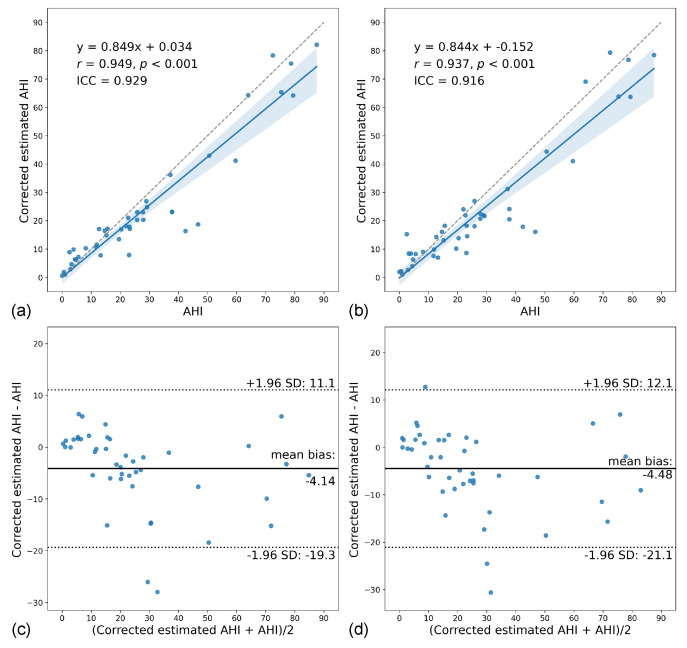
Comparison of the corrected estimated AHI with the ground truth for the binary (**a**,**c**) and multiclass models (**b**,**d**). (**a**,**b**) Scatter plots of corrected estimated AHI versus AHI showing the linear regression line (blue line) with 95% confidence intervals (blue shadowed area). (**c**,**d**) Bland–Altman plots of corrected estimated AHI and AHI.

The estimated AHI (Figure 7) and corrected estimated AHI (Figure 8) showed strong correlations (*r* = 0.805–0.949) and good to excellent agreement (ICC = 0.776–0.929) with the ground truth, with better results for the corrected. In the Bland–Altman analysis, the estimated AHI showed a mean bias of 1.60 (95% CI [−4.23, 7.43]) and 95% limits of agreement (LOA) of −36.0 to 39.2 for the binary model and a mean bias of 0.95 (95% CI [−4.19, 6.09]) and 95% LOA of −32.2 to 34.1 for the multiclass model. On the other hand, the corrected estimated AHI showed significantly lower values compared to the ground truth, with a mean bias of −4.14 (95% CI [−6.50, −4.23]) and 95% LOA of −19.3 to 11.1 for the binary model and a mean bias of −4.48 (95% CI [−7.06, −1.91]) and 95% LOA of −21.1 to 12.1 for the multiclass model.

**Figure 9:**
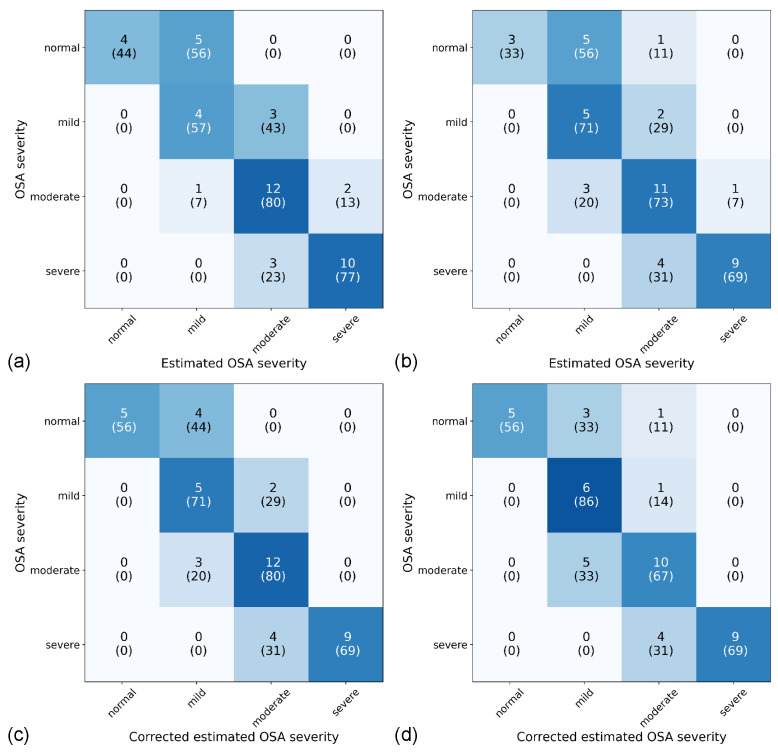
Confusion matrices for the estimation of OSA severity of the binary (**a**) and multiclass (**b**) models and the corrected estimated OSA severity of the binary (**c**) and multiclass (**d**) models. Data are presented as the number of participants with the row-normalized percentage in parentheses. The colors of the cells correspond to the row-normalized percentage.

The estimated OSA severities showed substantial agreement with the ground truth for both binary (κ = 0.715) and multiclass (κ = 0.648) models. The OSA severities estimated from the corrected estimated AHI showed a slightly higher substantial agreement with the ground truth for both the binary (κ = 0.736) and multiclass (κ = 0.699) models (Figure 9).

## 4. Discussion

With the increasing interest in non-contact sensors for PSG, radar constitutes a promising technology. The radar for sleep assessment primarily targets respiratory efforts, usually measured by thoracoabdominal belts in conventional PSG. However, there is scarce literature regarding the use of radars in OSA. Therefore, this preliminary study investigated the feasibility of the automated detection of apnea-hypopnea events for OSA diagnosis based on FMCW radar using deep learning.

The diagnostic performance was the highest for patients with severe OSA in terms of AUROC for per-segment classification and sensitivity for global event detection, consistent with previous studies using thoracoabdominal belts or radar [40,41,42,43]. This may be attributed to the higher proportion of apnea in patients with more severe OSA. While the sensitivities for hypopnea were not significantly different among the OSA severity groups, the proposed models showed higher sensitivities for apnea than hypopnea. In addition, our dataset included more patients with moderate or severe OSA than those without or with mild OSA. Therefore, because a much higher number of events were included in patients with more severe OSA, these events would have contributed more to the model training.

The proposed models showed limited sensitivities for hypopnea, unlike apnea, with a range of 47.6–58.1%, even though hypopnea was the major event (71.0%, 4427/6239). This is expected and concordant with previous studies [40,41,42,43], since hypopnea is an incomplete form of respiratory abnormality between normal respiration and the complete cessation of the upper airway (obstructive apnea). The diagnostic criteria for hypopnea include a more subtle change in airflow than apnea and require additional information on oxygen desaturation or arousal [32], which was not provided in the single-channel input of our study. Furthermore, lower sensitivities for hypopnea can be linked to more vulnerable interference with non-pathologic respiratory signals from changing body positions, limb movements, or sensor noise.

**Figure 10:**
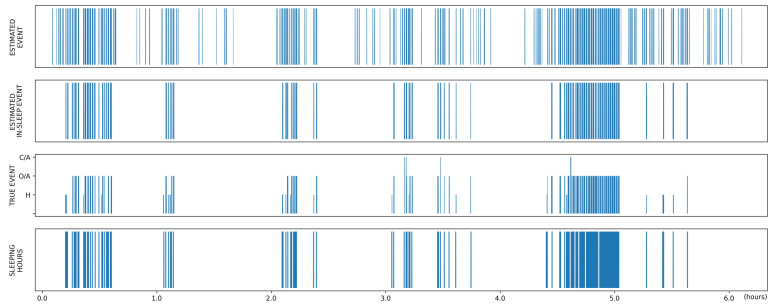
Prediction result of a whole PSG recording from an example case with poor sleep efficiency. The first row shows the estimated abnormal respiratory events. When considering sleep status (last row), false-positive detections at waking hours are reduced (second row), resulting in predictions more similar to the ground truth (third row). H = hypopnea, O/A = obstructive apnea, C/A = central apnea.

Most studies on OSA exclude data from waking hours, even when the sensor (e.g., thoracoabdominal belt) cannot directly determine the sleep status [40,41,42,43]. However, we included the entire PSG recording to simulate sleep monitoring environments where only radar was used and where it was incorporated with PSG. In the former, the estimated AHI showed strong correlations (*r* = 0.805–0.829) and good agreement with the ground truth (ICC = 0.776–0.812), and there was substantial agreement between the estimated and ground truth OSA severity (κ = 0.648–0.715). These results demonstrated the potential of the standalone use of radar for OSA screening. When considering only in-sleep estimated events, there were improvements in the estimation of AHI (*r* = 0.937–0.949 and ICC = 0.916–0.929) and OSA severity (κ = 0.699–0.736), primarily due to the reduction in false-positive detections during wakefulness. This is clearly shown in the case with poor sleep efficiency (Figure 10), which is an outlier in Figure 7.

We employed a detection model based on a CRNN architecture instead of the classification models used in most existing deep learning methods for OSA diagnosis [44]. The classification model has certain disadvantages. First, it uses weak labels compared to detection model. Previous works converted each segment of approximately 30 s to 1 min into a single class by thresholding the length of the in-segment event annotations [40,41]. However, the events manually annotated in PSG are strong labels, usually with a high temporal resolution of approximately 1 s, which can be exploited more by a detection model without losing information than a classification model. Second, a detection model provides more intuitive localization results for event detection than a classification model (Figure 6). The classification model requires postprocessing with a narrow sliding window to translate predictions into time-sequenced events (e.g., valid events if at least six consecutive segments are predicted to be abnormal) [43,45]. However, such postprocessing relies on a smoothing parameter that requires additional tuning after training the model.

We experimented with binary and multiclass models to investigate the potential benefits of multiclass labels in detecting abnormal events. However, they showed similar results, which may be attributed to several factors. First, there was a class imbalance in respiratory events. Hypopnea accounted for the majority of the events (71.0%, 4427/6239), followed by obstructive apnea (26.6%, 1660/6239), with central apnea being the fewest (2.4%, 152/6239). Second, because a multiclass task is more complicated than a binary task, multiclass labels may not necessarily help the model to learn to distinguish between abnormal and normal respiration. The multiclass model may learn the distributions of normal respiration in the early training phase at a level similar to that of the binary model, with the rest of the training focusing on distinguishing different classes of events.

This study had several limitations that should be addressed in future work. First, the number of study participants, especially those with less than moderate OSA, is insufficient. In addition, future studies should investigate more technical modifications of radar setups, such as signal processing or adding an under-the-mattress radar, and model development, including hyperparameter optimization and the comparison of different deep learning architectures. Specifically, advanced deep learning methods such as the Transformer [46] and deep domain adaptation [47] are potential alternatives. Moreover, regarding PSG, sleep stages (e.g., REM and non-REM), body position, and limb movements could be considered for model development and analysis. In particular, since sleep stages and body positions affect apnea-hypopnea frequency [48], future work could include the analysis of the effects of different sleep stages and body positions on diagnostic performances. In addition, a promising direction for future research is using an ensemble of the current model and models performing radar-based predictions of sleep stages and/or body movements.

In conclusion, we demonstrated that the automated detection of apnea-hypopnea events based on FMCW radar is feasible using a CRNN-based deep learning model. This preliminary study, which involved an OSA cohort still in recruitment and an in-development radar, showed the possibility of using the FMCW radar as a standalone screening tool for OSA. Integration with other non-contact sensors for sleep signals, such as oxygen desaturation sensors, is warranted to develop an improved non-contact OSA diagnosis.

## Figures and Tables

**Table 1:** Demographic and polysomnographic data of the study population.

Variables		Normal	Mild OSA	Moderate OSA	Severe OSA
Subject characteristics	Number (male/female)	9 (2/7)	7 (4/3)	15 (10/5)	13 (9/4)
	Age	46.4 ± 17.9	49.9 ± 18.0	57.9 ± 9.5	55.3 ± 17.5
	Body mass index (kg/m^2^)	24.6 ± 4.1	24.9 ± 1.9	25.2 ± 2.9	26.7 ± 17.5
	Neck circumference (cm)	36.7 ± 3.0	39.0 ± 3.6	38.6 ± 4.2	40.8 ± 3.1
	ESS score	4.8 ± 3.1	5.0 ± 3.3	9.0 ± 3.7	8.5 ± 3.8
	SSS score	2.2 ± 1.0	3.0 ± 0.8	2.0 ± 0.5	2.4 ± 0.8
	PSQI score	8.6 ± 3.2	11.0 ± 2.8	10.0 ± 3.5	9.7 ± 4.0
Polysomnographic data	Time in bed (min)	362.6 ± 106.5	412.0 ± 16.2	396.0 ± 23.9	400.1 ± 24.4
	Total sleep time (min)	294.4 ± 97.2	311.0 ± 43.6	314.8 ± 66.7	266.5 ± 90.6
	Sleep latency (min)	18.2 ± 30.5	17 ± 12.7	16.4 ± 27.6	13.8 ± 14.8
	Sleep efficiency (%)	80.1 ± 8.7	75.7 ± 11.7	78.9 ± 13.7	66.3 ± 22.1
	N1 (%)	12.3 ± 7.2	14.0 ± 2.6	16.9 ± 8.0	33.4 ±13.5
	N2 (%)	52.3 ± 5.0	51.4 ± 5.7	51.3 ± 6.4	47.2 ± 9.6
	N3 (%)	20.0 ± 3.0	19.2 ± 2.1	16.7 ± 5.3	8.5 ± 8.4
	REM (%)	15.4 ± 9.6	15.4 ± 6.7	15.0 ± 6.2	10.9 ± 7.9
	Apnea index (events/h)	0	0.1 ± 0.2	2.8 ± 3.2	20.1 ± 20.6
	Hypopnea index (events/h)	2.5 ± 1.9	10.3 ± 2.6	20.3 ± 4.2	34.6 ± 11.0
	AHI (events/h)	2.6 ± 1.7	11.1 ± 3.1	23.3 ± 4.4	59.2 ± 18.2
	RERA index (events/h)	0.1 ± 0.3	0	0	0
	Arousal index (events/h)	19.9 ± 10.7	20.8 ± 5.9	29.4 ± 10.4	53.9 ± 17.6
	Lowest O2 saturation (%)	89.0 ± 3.3	87.7 ± 2.6	83.6 ± 3.4	72.8 ± 11.2
Number of segments	Abnormal	296	906	3917	6016
	Normal	6298	4911	8063	4485

Data are presented as the mean ± standard deviation. N1, N2, N3, and REM sleep stages. OSA, obstructive sleep apnea; ESS, Epworth Sleepiness Scale; SSS, Stanford Sleepiness Scale; PSQI, Pittsburgh Sleep Quality Index; AHI, apnea-hypopnea index; RERA, respiratory effort-related sleep arousal.

**Table 2:** Per-segment classification performance in the overall study population and OSA severity groups.

Model	Metric	Overall	Normal	Mild	Moderate	Severe
Binary	AUROC	0.846 [0.842, 0.851]	0.819 [0.793, 0.846]	0.796 [0.780, 0.812]	0.798 [0.789, 0.807]	0.859 [0.852, 0.866]
	Sensitivity	0.744 (8289/11135) [0.736, 0.753]	0.625 (185/296) [0.570, 0.680]	0.657 (595/906) [0.626, 0.688]	0.674 (2641/3917) [0.660, 0.689]	0.809 (4868/6016) [0.799, 0.819]
	Specificity	0.803 (19065/23757) [0.797, 0.808]	0.868 (5464/6298) [0.859, 0.876]	0.767 (3916/4911) [0.786, 0.809]	0.781 (6293/8063) [0.771, 0.790]	0.756 (3392/4485) [0.744, 0.769]
	PPV	0.639 (8289/12981) [0.630, 0.648]	0.182 (185/1019) [0.138, 0.226]	0.374 (595/1590) [0.343, 0.406]	0.599 (2641/4411) [0.583, 0.614]	0.817 (4868/5961) [0.807, 0.826]
	NPV	0.870 (19065/21911) [0.866, 0.874]	0.980 (5464/5575) [0.977, 0.984]	0.926 (3916/4227) [0.919, 0.934]	0.831 (6293/7569) [0.823, 0.840]	0.747 (3392/4540) [0.734, 0.760]
	Accuracy	0.784 (27354/34892) [0.780, 0.788]	0.857 (5649/6594) [0.848, 0.865]	0.776 (4511/5817) [0.765, 0.786]	0.746 (8934/11980) [0.738, 0.754]	0.787 (8260/10501) [0.779, 0.794]
Multiclass	AUROC	0.844 [0.840, 0.849]	0.816 [0.788, 0.843]	0.807 [0.791, 0.822]	0.795 [0.787, 0.804]	0.858 [0.850, 0.865]
	Sensitivity	0.721 (8024/11135) [0.712, 0.729]	0.628 (186/296) [0.573, 0.683]	0.605 (548/906) [0.573, 0.637]	0.638 (2498/3917) [0.623, 0.653]	0.797 (4792/6016) [0.786, 0.807]
	Specificity	0.813 (19305/23757) [0.808, 0.818]	0.850 (5352/6298) [0.841, 0.859]	0.829 (4070/4911) [0.818, 0.839]	0.800 (6454/8063) [0.792, 0.809]	0.765 (3429/4485) [0.752, 0.777]
	PPV	0.643 (8024/12476) [0.634, 0.652]	0.164 (186/1132) [0.122, 0.207]	0.395 (548/1389) [0.363, 0.426]	0.608 (2498/4107) [0.593, 0.624]	0.819 (4792/5848) [0.810, 0.829]
	NPV	0.861 (19305/22416) [0.857, 0.866]	0.980 (5352/5462) [0.976, 0.983]	0.919 (4070/4428) [0.912, 0.927]	0.820 (6454/7873) [0.811, 0.828]	0.737 (3429/4653) [0.724, 0.750]
	Accuracy	0.783 (27329/34892) [0.779, 0.788]	0.840 (5538/6594) [0.831, 0.849]	0.794 (4618/5817) [0.783, 0.804]	0.747 (8952/11980) [0.739, 0.755]	0.783 (8221/10501) [0.775, 0.791]

Data are presented as values (numerator/denominator, if applicable) [95% confidence intervals]. AUROC, area under the receiver operating characteristic curve; PPV, positive predictive value; NPV, negative predictive value.

**Table 3:** Global event detection performance in the overall study population and OSA severity groups.

Model	Metric	Group	Overall	Normal	Mild	Moderate	Severe
Binary	Sensitivity	Overall	0.633 (3948/6239) [0.621, 0.645]	0.583 (74/127) [0.497, 0.668]	0.551 (252/457) [0.506, 0.597]	0.573 (1123/1960) [0.551, 0.595]	0.676 (2499/3695) [0.661, 0.691]
		H	0.539 (2384/4427) [0.524, 0.553]	0.581 (72/124) [0.494, 0.668]	0.529 (220/416) [0.481, 0.577]	0.523 (894/1710) [0.499, 0.547]	0.550 (1198/2177) [0.529, 0.571]
		O/A	0.870 (1444/1660) [0.854, 0.886]	1.000 (2/2) [1.000, 1.000]	1.000 (5/5) [1.000, 1.000]	0.920 (207/225) [0.885, 0.955]	0.861 (1230/1428) [0.843, 0.879]
		C/A	0.790 (120/152 [0.725, 0.854]	0.000 (0/1) [0.000, 0.000]	0.750 (27/36) [0.609, 0.892]	0.880 (22/25) [0.753, 1.000]	0.789 (71/90) [0.705, 0.873]
	PPV		0.695 (3948/5681) [0.683, 0.707]	0.260 (74/285) [0.209, 0.311]	0.475 (252/531) [0.432, 0.517]	0.674 (1123/1666) [0.652, 0.697]	0.781 (2499/3199) [0.767, 0.796]
	FP/patient	Overall	39.0 [30.5, 47.6]	23.4 [18.4, 28.5]	39.9 [35.4, 44.3]	36.1 [32.3, 40.0]	52.8 [39.1, 66.4]
		In-sleep	26.0 [18.6, 33.4]	17.2 [13.1, 21.4]	28.0 [23.7, 32.3]	25.9 [22.2, 29.5]	31.0 [18.7, 43.4]
Multiclass	Sensitivity	Overall	0.622 (3883/6239) [0.610, 0.634]	0.543 (69/127) [0.457, 0.630]	0.490 (224/457) [0.444, 0.536]	0.557 (1092/1960) [0.535, 0.579]	0.676 (2498/3695) [0.661, 0.691]
		H	0.525 (2323/4427) [0.510, 0.539]	0.540 (67/124) [0.453, 0.628]	0.476 (198/416) [0.428, 0.524]	0.508 (868/1710) [0.484, 0.531]	0.547 (1190/2177) [0.526, 0.568]
		O/A	0.869 (1443/1660) [0.853, 0.886]	0.500 (1/2) [0.000, 1.000]	1.000 (5/5) [1.000, 1.000]	0.898 (202/225) [0.858, 0.937]	0.865 (1235/1428) [0.847, 0.883]
		C/A	0.770 (117/152) [0.703, 0.837]	1.000 (1/1) [1.000, 1.000]	0.583 (21/36) [0.422, 0.744]	0.880 (22/25) [0.753, 1.000]	0.811 (73/90) [0.730, 0.892]
	PPV		0.695 (3883/5585) [0.683, 0.707]	0.199 (69/347) [0.157, 0.241]	0.489 (224/458) [0.443, 0.535]	0.673 (1092/1622) [0.650, 0.696]	0.791 (2498/3158) [0.777, 0.805]
	FP/patient	Overall	38.5 [29.9, 47.0]	30.9 [23.7, 38.1]	33.4 [29.3, 37.5]	35.3 [30.3, 40.2]	50.1 [37.0, 63.2]
		In-sleep	25.3 [17.7, 32.8]	22.2 [16.2, 28.3]	24.1 [20.4, 27.9]	24.7 [20.0, 29.3]	28.7 [16.6, 40.8]

Data are presented as value (numerator/denominator, if applicable) [95% confidence intervals]. PPV, positive predictive value; FP, false-positive detection; H, hypopnea; O/A, obstructive apnea; C/A, central apnea.

## Data Availability

Not applicable.

## Supplementary Materials

The following supporting information can be downloaded at: https://www.mdpi.com/article/10.3390/s22197177/s1. Figure S1: The signal processing flows in the FMCW radar to detect (a) distance and (b) velocity of the target.
