# Supplementary material for: Automated Detection of Sleep Apnea-Hypopnea Events Based on 60 GHz Frequency-Modulated Continuous-Wave Radar Using Convolutional Recurrent Neural Networks: A Preliminary Report of a Prospective Cohort Study
Source: Sensors (Basel). 2022 Sep 21;22(19):7177. doi: 10.3390/s22197177 (PMC9570824; doi:10.3390/s22197177)
Supplement: Supplementary file 1 [file sensors-22-07177-s001.zip › sensors-1898705-supplementary.pdf]

Supplementary Materials

An FMCW radar transmits and receives signals to simultaneously detect the distance and velocity of the target. The radar launches a chirp signal with a linearly increasing frequency from 60 GHz to 64 GHz. Then, the signal is reflected by the target and returns to the radar, where it is mixed with a locally oscillating 60 GHz signal. The time difference between the transmitted and received signals produces a sinusoidal wave. The radar computes the distance and velocity of the target based on sinusoidal waves acquired from each chirp. The output wave from a single chirp was converted to the target distance by applying a fast Fourier transform (FFT) (Figure S1a). The velocity was detected by conducting FFT to determine the phase differences between the waves from consecutive chirps (Figure S1b).

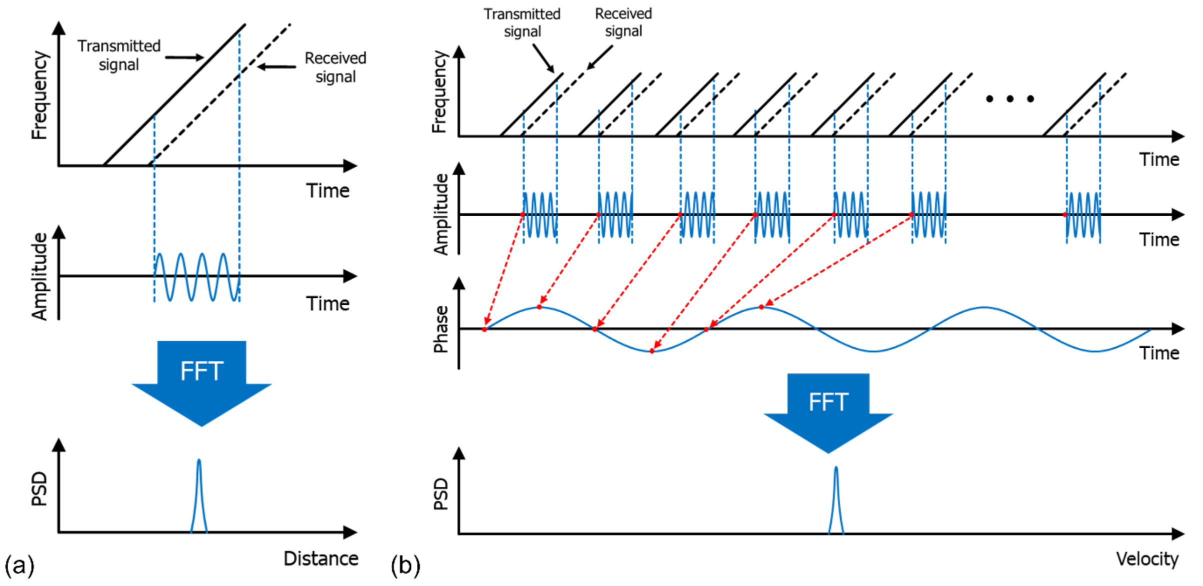

Figure S1. The signal processing flows in the FMCW radar to detect (a) distance and (b) velocity of the target.
